# Microimaging: Seeing the Unseen in Living Patients

**DOI:** 10.1155/2014/797108

**Published:** 2014-12-30

**Authors:** Guillermo J. Tearney

**Affiliations:** Wellman Center for Photomedicine, Massachusetts General Hospital and Harvard Medical School, USA

## Abstract

Today's gold standard for medical diagnosis is histology of excised biopsies or surgical specimens where tissue is taken out of the body, processed, sectioned, stained, and looked at under a light microscope by a pathologist. There are many limitations of this technique, including the fact that it is inherently invasive, time consuming, costly, and dangerous for some organs. Furthermore, oftentimes the diseased tissue is not readily seen by visual inspection and as a result the tissue is sampled at a random location, which can be highly inaccurate. If we could instead conduct microscopy inside the body, then we could provide tools for screening, targeting biopsies, making primary disease diagnosis, and guiding intervention on the cellular basis. This promise has motivated the development of a new field, termed in vivo microscopy, the goal of which is to obtain microscopic images from living human patients. Two in vivo microscopy technologies, confocal microscopy and optical coherence tomography, are currently available and in clinical use. Upcoming developments, including whole organ microscopy, swallowable microscopy capsules, molecular imaging, and very high resolution microscopic devices, are in the pipeline and will likely revolutionize how disease is diagnosed and how medicine is practiced in the future.

